# Towards Personalized Medicine Mediated by *in Vitro* Virus-Based Interactome Approaches

**DOI:** 10.3390/ijms15046717

**Published:** 2014-04-21

**Authors:** Hiroyuki Ohashi, Etsuko Miyamoto-Sato

**Affiliations:** Division of Interactome Medical Sciences, the Institute of Medical Science, the University of Tokyo, 4-6-1 Shirokanedai, Minato-ku, Tokyo 108-8639, Japan; E-Mail: ohashi@yokohamalken.or.jp

**Keywords:** cell-free protein synthesis system, *in vitro* selection, *in vitro* virus, protein-protein interaction

## Abstract

We have developed a simple *in vitro* virus (IVV) selection system based on cell-free co-translation, using a highly stable and efficient mRNA display method. The IVV system is applicable to the high-throughput and comprehensive analysis of proteins and protein–ligand interactions. Huge amounts of genomic sequence data have been generated over the last decade. The accumulated genetic alterations and the interactome networks identified within cells represent a universal feature of a disease, and knowledge of these aspects can help to determine the optimal therapy for the disease. The concept of the “integrome” has been developed as a means of integrating large amounts of data. We have developed an interactome analysis method aimed at providing individually-targeted health care. We also consider future prospects for this system.

## 1. Introduction

It has been over 10 years since the human genome sequence was decoded by the Human Genome Project. Personal genome analysis based on post-genome functional analysis and multi-omics analysis of personal medical information has been identified as the future way forward. However, the advent of next-generation sequencing (NGS), the clinical application of the technology, the quality of the technology available for analyzing multi-omics data for personalized medical care, the cost, and the amount of data available have become issues. Interactome analysis is an important aspect of multi-omics analysis. The concept of the “integrome” has been developed as a means of integrating large amounts of data. We have developed an interactome approach aimed at individualized health care, and discuss the future prospects for this technology.

## 2. Comprehensive Protein–Protein Interaction Analysis in Post-Genome Analysis

Elucidating protein functionality is a challenge of the post-genomic era, and much research has focused on trying to understand the relevance of protein structure and function. Studying protein–protein interactions (PPIs) also provides a means of analyzing the function of the relationships, and methods of comprehensively analyzing PPIs have been developed. The yeast two hybrid (Y2H) [1] and affinity purification-mass spectrometry (AP-MS) methods [2] are established PPI analysis tools that have been responsible for generating large amounts of PPI data (Table 1). However, the data produced by these techniques have very high levels of false positives and false negatives. For example, the exact rate of false positive results with Y2H experiment is not known, but earlier estimates were as high as 70% [3]. False positive rates for AP experiments could be as high as 77% [4].

We have developed an *in vitro* virus (IVV) system [5,6] as a PPI analysis tool. IVV involves covalent binding of an mRNA molecule and the protein encoded by the mRNA through puromycin. The IVV is synthesized from cDNA using a cell-free translation system, thus avoiding the issue of biological toxicity happens in cells. This method allows the acquisition of more data compared with cell-based experimental systems and can compensate for the disadvantages of AP-MS and Y2H, thus providing complementary PPI data.

*In vitro* selection experiments using mRNA display methods such as IVV [6,7] or mRNA-peptide fusions [8–10], which were originally developed for evolutionary protein engineering, are expected to be powerful tools for analyzing protein functions in the post-genomic era [11,12]. mRNA display also represents a potentially useful method if adapted for high-throughput *in vitro* analysis of PPIs and complexes [12]. mRNA display is composed of four essential processes: transcription, translation, selection, and reverse transcription-polymerase chain reaction (RT-PCR). A stable and efficient IVV [6] should allow simple selection without any requirement for post- translational processes.

Furthermore, we have developed a new method for labeling proteins, which will also be useful for PPI analysis [5,13,14]. Generally, site-specific fluorescent labeling of proteins using conventional chemical methods is difficult. However, puromycin-labeling of the *C*-terminus of the full-length protein can be done easily, simultaneously with protein synthesis, in a cell-free translation system. By adjusting the concentration of puromycin, proteins can be labeled without impairing their original functions. We confirmed the known PPI between protein A and human IgG [15], previously determined by fluorescence polarization assay, using *C*-terminus-labeling technology [13]. Moreover, labeling of the *C*-terminus of the protein using this new method is also useful for improving the accuracy of molecular-selectivity testing after IVV selection. PPI analysis using methods such as the two-hybrid method [1,16,17] results in a proportion of false-positives, and minimizing the incidence of false positives is important for obtaining biologically-relevant data. *In vitro* post-selection can reduce the occurrence of false positives and provide information about direct/indirect interactions. Post-selection comprises a pull-down assay to confirm the interactions using our *C*-terminal protein-labeling method [5,6,13,14]. The use of post-selection should thus provide reliable data for PPI analysis.

## 3. Interactome Analysis Using Next-Generation Sequencing in the Personal-Genome Era

The advent of NGS has dramatically increased the availability of large-scale data for use in personal genomics [18–20]. However, the completeness of PPI data remains poor [21,22]. For example, it is estimated that less than 10% of the human interactome has been identified to date [21]. Interactome analysis using NGS and Y2H has improved the completeness of the data compared with the conventional method using the Sanger sequencing [23,24], but coverage is limited by cytotoxicity. In addition, the Y2H method generates false-positive results for interactions, and this problem is not improved by NGS.

**Figure 1.**
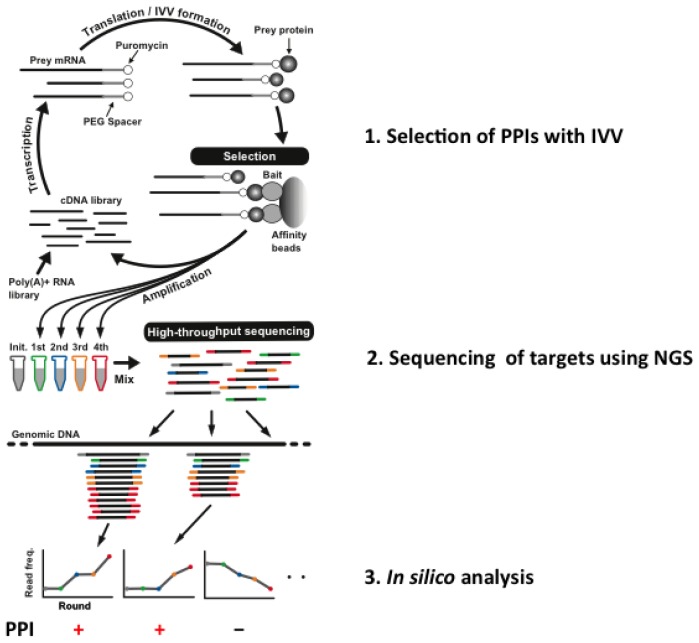
Overview of *in vitro* virus (IVV)-HiTSeq, a cell-free system for detecting interactors with target bait proteins.

The IVV-HiTSeq (high-throughput sequencing) method [25], which is a combination of NGS and IVV, has been developed with the aim of overcoming these problems. Selections using the IVV method are conducted under cell-free conditions [12], and subsequent sequencing by NGS is not limited by cloning steps using any kind of cells (Figure 1).

The IVV-HiTSeq method thus has the potential to produce large amounts of accurate protein-interaction data. The cell-free aspect of the experimental procedure is one of the main advantages allowing the highly-efficient production of interaction data. The combination of IVV and high-throughput sequencing does not require any host cells for DNA cloning; a step that previously limited the efficiency of screening and the number of interactions that could be examined. In addition, the IVV method can select from a cDNA library consisting of 10^12^ molecules, which is beyond the capacity of conventional high-throughput protein-selection methods (Table 1) [26,27], and coverage of the interactome is expected to increase in line with further expected increases in NGS throughput. Notably, the completely cell-free procedure will also allow the analysis of cytotoxic proteins, leading to a more comprehensive interactome analysis. With respect to the accuracy of IVV-HiTSeq data, the use of library-specific barcoded primers and *in silico* analysis reduces the number of false positive interactions contained in the initial raw data [25]. IVV-HiTSeq was compared with conventional IVV using Sanger sequencing for the same prey library and bait, and 640 sequences (87%) determined by Sanger sequencing were also obtained by IVV-HiTSeq; however, most of the sequences (99.7%) obtained by IVV-HiTSeq were new and not found by Sanger sequencing. Moreover, 88% of the real-time PCR assays that were followed by IVV-HiTSeq, including *in silico* analysis, were positive. IVV-HiTSeq has the potential to provide verification data comparable to real-time PCR assays, and could generate data equivalent to several thousands of real-time PCR confirmations, resulting in reductions in both cost and time. IVV-HiTSeq also has the ability to reproduce data and to reduce false negatives, compared with the conventional method using Sanger sequencing. Importantly, however, the method dramatically reduces the incidence of false positives [25]. Researchers in the fields of cellular biology and physiology will therefore be able to have more confidence in the interaction data generated by IVV-HiTSeq compared with data obtained using conventional methods. IVV-HiTSeq is potentially applicable to many cell-free display technologies, such as mRNA display, DNA display, and ribosome display. Moreover, IVV can be applied not only to the *in vitro* selection of PPIs, but also to the detection of protein–DNA, protein–RNA and protein–chemical compound interactions [28], suggesting that IVV-HiTSeq could become a universal tool for exploring protein sequences and interaction networks.

A cDNA library is initially created from poly(A) + RNAs by random priming. The cDNA is then transcribed into mRNA, and polyethylene glycol (PEG) + puromycin spacers are ligated to their 3′ ends. mRNA-protein molecules, linked via puromycin, are formed during *in vitro* translation. Prey molecules that interact with tagged bait proteins are then captured by affinity beads and purified. The mRNA moieties of selected prey molecules are amplified by RT-PCR using two types of primers; one for the next selection round and another for high-throughput sequencing. The second type of primer contains a barcoded region (indicated in grey, green, blue, yellow and red), with four selection-round-specific bases. The reads generated by high-throughput sequencing are sorted by their barcoded parts and mapped to known genomic sequences. Read frequencies for each genomic position are calculated for each selection round and used to determine the enriched regions. To use of the barcoded primers can reduce a risk of cross contamination between libraries. Also, greater sequencing depth can be helpful for a PPI analysis to avoid a contamination between samples. Roche 454 Sequencer was used for the experiment. Statistical significance is calculated by comparing the read frequencies with the frequencies of the initial library and the negative control.

## 4. Future Prospects for Interactome Analysis in Personal Genomics

**Figure 2.**
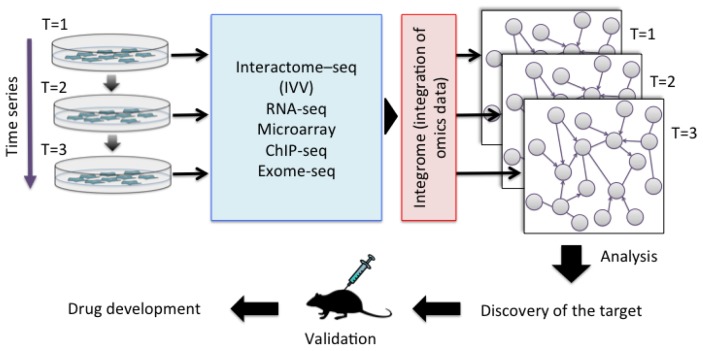
Integration of multi-omics data in the process of personalized medicine.

The accumulation of genetic alterations and the interactome networks in cancer cells represent a universal feature of the disease, and knowledge of these factors can help to determine the optimal therapy for the disease. Various cancer-related proteins have been identified and their *in vivo* functions have been revealed. However, understanding the function of a particular protein is not enough [29], because cancer involves complex gene pathways *in vivo*. An individual approach is therefore essential for understanding the personality of a cancer. Cancer not only differs between people, but also changes within the same individual over time. Future understanding of the personality of cancer cells will require the collection of data by multi-omics analysis. Multi-omics includes gene-, transcription-, and protein-specific information. In contrast, the interactome includes network data based on direct interactions between molecules. An integrated approach including both interactome and multi-omics data is therefore needed to compare the identities of cancer cells. This approach is referred to as “integrome analysis” [30]. The integrome is a network map of the interactome together with a list of multi-omics data, which will allow the analysis of differences between cancer cells and normal cells [31], the effects of treatment, and important factors such as biomarkers (Figure 2). Since PPI is at the core of the biomolecule network, we have developed IVV system to detect PPIs toward personal genomics. We have succeeded to obtain significant results of interactome analysis by using the system. On the other hand, the system has a limitation in the high-throughput screening and identification of interaction pairs of proteins, due to the time consuming preparation of bait proteins and the low ability of the conventional sequencing method. Recently, a method antibody for a selection with a bicistronic IVV system relies on *in vitro* compartmentalization in water-in-oil emulsions was reported [32]. Using the system, man-made cell-like compartments make it possible to display oligomeric proteins in a cell-free translation system, without preparation of baits. We are also trying to develop “a bait-free IVV”, but for a whole-cell analysis, which enables all genes encoding interacting protein pairs to be linked.

An advantage of IVV is the size of large library size (up to 10^12^). The use of next-generation sequencing will be able to maximize the potential of IVV. The latest Roche 454 Sequencer can sequence approximately 10^6^ reads of approximately 1000 bp per one run, long enough to cover both of the linked variable regions. 10^6^ reads are not enough to cover the whole selected IVV library, may make it possible to obtain unique high affinity binders. Further possibilities may be considered when the specifications of the next generation sequencers improve even more. The speed of improvement for this technology is remarkable, and when it becomes possible to sequence the whole selected IVV library, it should allow selection of low affinity ligands that would usually be lost in a typical selection of repetitious rounds. IVV libraries will be subjected to high-throughput sequencing by NGS to generate interactome information. This will facilitate archiving of the interactome map of a whole-cell library at low cost. We suggest that IVV systems can provide an important contribution to our understanding of the interactome networks in cancer cells, and thus help in the development of pharmaceutical agents to treat currently intractable diseases.

The dynamics of the pharmacological response mechanisms can be examined by analyzing the integrated multi-omics data. First, the time series of interactome–seq (IVV), RNA-seq, microarray, ChIP-seq and exome-seq are integrated. Second, an efficient module-detecting algorithm is applied to the composite maps. The maps can then be used to compare cancer cells and normal cells, and to assess the effects of medicines. Finally, the identified targets can be validated in animal experiments for subsequent drug development.

## Figures and Tables

**Table 1.** Comparison of comprehensive protein–protein interaction analysis methods.

Method	Experimental system	Library size	Cell cloning required	Next generation sequencing
Y2H^a^	*In vivo*	10^6^	Yes	Applicable, but limited
AP-MS^b^	*In vivo*	-	Yes	Inapplicable
IVV	*In vitro*	10^12^	No	Applicable and effective

a Y2H: yeast two hybrid;

b AP-MS: affinity purification-mass spectrometry.
